# Microbiomes Detected by Bronchoalveolar Lavage Fluid Metagenomic Next-Generation Sequencing among HIV-Infected and Uninfected Patients with Pulmonary Infection

**DOI:** 10.1128/spectrum.00005-23

**Published:** 2023-07-12

**Authors:** Yuting Tan, Zhong Chen, Ziwei Zeng, Songjie Wu, Jie Liu, Shi Zou, Min Wang, Ke Liang

**Affiliations:** a Department of Infectious Diseases, Zhongnan Hospital of Wuhan University, Wuhan, China; b Wuhan Research Center for Infectious Diseases and Cancer, Chinese Academy of Medical Sciences, Wuhan, China; c Department of Infection and Immunology, The First Hospital of Changsha City, Changsha, China; d Graduate Collaborative Training Base of the First Hospital of Changsha, Hengyang Medical School, University of South China, Hengyang, China; e Department of Nosocomial Infection Management, Zhongnan Hospital of Wuhan University, Wuhan, China; f Hubei Engineering Center for Infectious Disease Prevention, Control and Treatment, Wuhan, China; The University of Auckland

**Keywords:** pulmonary infection, mNGS, bronchoalveolar fluid, HIV, human immunodeficiency virus

## Abstract

Comparison of lung microbiomes between HIV-infected and uninfected patients with pulmonary infection by metagenomic next-generation sequencing (mNGS) has not been described in China. The lung microbiomes detected in bronchoalveolar fluid (BALF) by mNGS among HIV-infected and uninfected patients with pulmonary infection were reviewed in the First Hospital of Changsha between January 2019 and June 2022. In total, 476 HIV-infected and 280 uninfected patients with pulmonary infection were enrolled. Compared with HIV-uninfected patients, the proportions of Mycobacterium (*P* = 0.011), fungi (*P* < 0.001), and viruses (*P* < 0.001) were significantly higher in HIV-infected patients. The higher positive rate of Mycobacterium tuberculosis (MTB; *P* = 0.018), higher positive rates of Pneumocystis jirovecii and *Talaromyces marneffei* (all *P* < 0.001), and higher positive rate of cytomegalovirus (*P* < 0.001) contributed to the increased proportions of Mycobacterium, fungi, and viruses among HIV-infected patients, respectively. The constituent ratios of Streptococcus pneumoniae (*P* = 0.007) and Tropheryma whipplei (*P* = 0.002) in the bacteria spectrum were significantly higher, while the constituent ratio of Klebsiella pneumoniae (*P* = 0.005) was significantly lower in HIV-infected patients than in HIV-uninfected patients. Compared with HIV-uninfected patients, the constituent ratios of *P. jirovecii* and *T. marneffei* (all *P* < 0.001) in the fungal spectrum were significantly higher, while the constituent ratios of *Candida* and Aspergillus (all *P* < 0.001) were significantly lower in HIV-infected patients. In comparison to HIV-infected patients without antiretroviral therapy (ART), the proportions of T. whipplei (*P* = 0.001), MTB (*P* = 0.024), *P. jirovecii* (*P* < 0.001), *T. marneffei* (*P* < 0.001), and cytomegalovirus (*P* = 0.008) were significantly lower in HIV-infected patients on ART. Significant differences in lung microbiomes exist between HIV-infected and uninfected patients with pulmonary infection, and ART influences the lung microbiomes among HIV-infected patients with pulmonary infection.

**IMPORTANCE** A better understanding of lung microorganisms is conducive to early diagnosis and treatment and will improve the prognosis of HIV-infected patients with pulmonary infection. Currently, few studies have systematically described the spectrum of pulmonary infection among HIV-infected patients. This study is the first to provide comprehensive information on the lung microbiomes of HIV-infected patients with pulmonary infection (as assessed by more sensitive metagenomic next-generation sequencing of bronchoalveolar fluid) compared with those from HIV-uninfected patients, which could provide a reference for the etiology of pulmonary infection among HIV-infected patients.

## INTRODUCTION

The respiratory tract is the most frequently infected site in individuals with HIV (1). Pulmonary infection is not only one of the leading causes of morbidity and mortality but also one of the most common causes of hospitalization among HIV-infected patients (2, 3). Differences in pulmonary infection exist between HIV-infected and uninfected patients, where opportunistic infections are more common in the former due to severe cellular immune dysfunction. However, because of the limited sensitivity of conventional microbiological tests (smear, culture, PCR, etc.) observed in clinical practice, it is still a challenge to fully characterize the pathogens contributing to pulmonary infection in HIV-infected patients. A better understanding of lung microbiomes is conducive to early diagnosis, providing benefits for early treatment and improving the prognosis among HIV-infected patients with pulmonary infection.

In recent years, metagenomic next-generation sequencing (mNGS; a high-throughput sequencing technique of all the nucleic acids from pathogenic specimens, commensals, and hosts) is increasingly used in diagnosing different infectious diseases, including pneumonia (4), central nervous system infection (5), and bloodstream infection (6). Conventional microbiological tests are limited by low positive detection rates, poor timeliness, and less acquisition of pathogen information. By contrast, mNGS can rapidly and comprehensively identify pathogens, including rare ones, thereby facilitating early diagnosis and prompt treatment of infectious diseases (7). In both immunocompetent and immunosuppressed populations, mNGS has been reported to have significantly higher sensitivity than conventional methods in identifying pathogens causing pulmonary infections (4, 8).

In HIV-infected patients, the number of case reports that identify pathogens of different infectious diseases by mNGS has increased in recent years (9–13). However, so far, there has been no study investigating and summarizing the lung microbiome spectra among HIV-infected patients with pulmonary infection by mNGS. Here, we conduct a retrospective cohort study to investigate the lung microbiomes of HIV-infected patients with pulmonary infection compared with those of uninfected patients with pulmonary infection as assessed by mNGS of bronchoalveolar fluid (BALF).

## RESULTS

### Patient characteristics.

**FIG 1:**
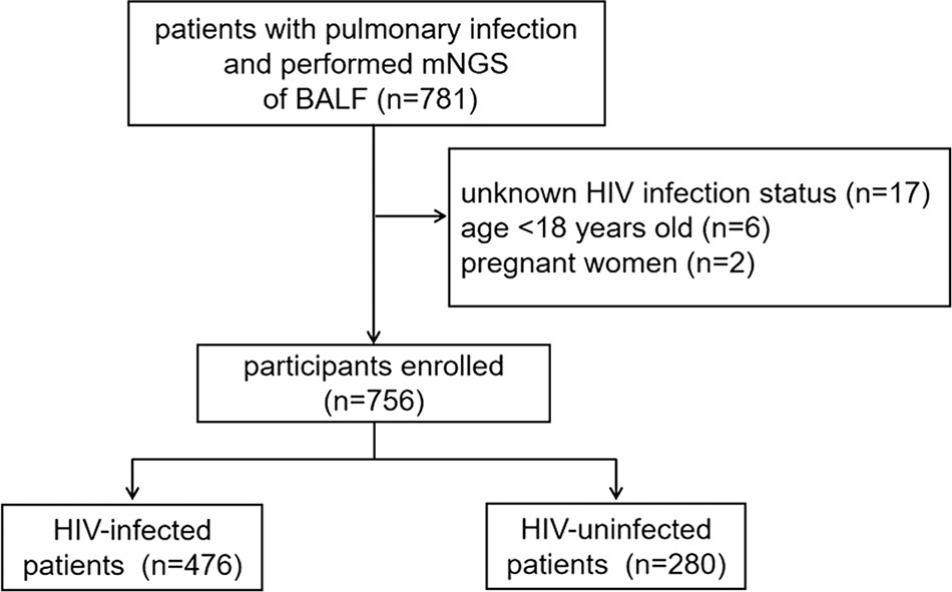
Flowchart of study participants enrolled in our study.

In total, 781 patients with pulmonary infection that had mNGS performed on their BALF were recruited in our study. Out of the 781 patients, 17 had unknown HIV infection status, 6 were under 18 years old, and 2 were pregnant women. A final number of 756 patients were enrolled in our study. The flowchart of study participant enrollment is shown in Fig. 1.

Of the 756 patients with pulmonary infection, 476 were infected with HIV, and 280 were uninfected. The median age of HIV-infected patients was younger than that of uninfected individuals (*P* < 0.001). Among HIV-infected patients, the proportion of males was significantly higher (*P* < 0.001), and the proportions of antibiotics use (*P* = 0.049) and lymphocyte counts (*P* < 0.001) were significantly lower than those among uninfected individuals. Characteristics of HIV-infected and uninfected patients with pulmonary infection are shown in Table 1.

**TABLE 1:** Characteristics of patients with pulmonary infection comparing those with HIV infection to those without HIV infection

Patient characteristics^*a*^	HIV-infected patients	HIV-uninfected patients	*P* value
	(*n* = 476)	(*n* = 280)	
Age (yrs, median [IQR])	49 (37 to 58)	65 (54 to 75)	<0.001
Male, *n* (%)	397 (83.4)	176 (62.8)	<0.001
On ART, *n* (%)	212 (44.5)	/^*b*^	/^*b*^
Antibiotic usage within 3 mo, *n* (%)	452 (94.9)	274 (97.8)	0.049
Immunosuppressive therapy use within 3 mo, *n* (%)	179 (37.6)	99 (35.3)	0.536
HIV load (copies/mL, median [IQR])	180,000 (39,150 to 654,250)	/^*b*^	/^*b*^
Lymphocyte counts (per μL, median [IQR])	725 (442 to 1,157)	1070 (780 to 1,640)	<0.001
CD4^+^ T cell count (per μL, median [IQR])	50 (20 to 149)	/^*b*^	/^*b*^

a IQR, interquartile range.

b /, no data.

### Lung microbiomes detected by mNGS.

**FIG 2:**
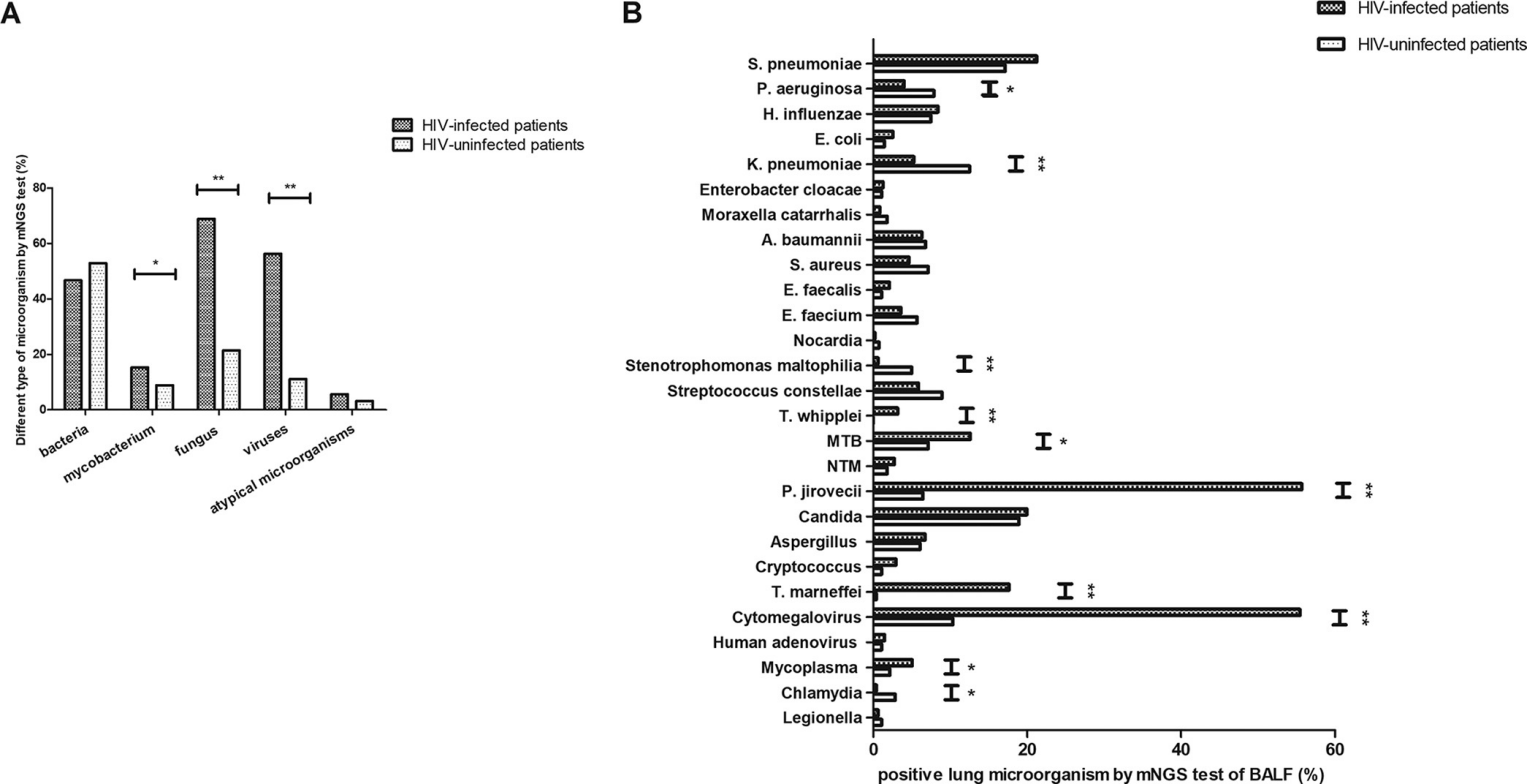
(A) Different types of microbiomes detected by mNGS of BALF among HIV-infected and uninfected patients with pulmonary infection. (B) Comparison of lung microbiomes detected by mNGS of BALF between HIV-infected and HIV-uninfected patients with pulmonary infection; *, *P* < 0.05; **, *P* < 0.01.

As detected by mNGS of BALF, fungi (68.9%) and viruses (56.3%) were the most common microorganisms in the lung microbiomes of 476 HIV-infected patients with pulmonary infection, followed by bacteria (46.6%) (Fig. 2A). By contrast, bacteria (52.8%) and fungi (21.4%) were the most frequently detected microorganisms in lung microbiomes of 280 HIV-uninfected patients with pulmonary infection, followed by viruses (11.1%). Compared with HIV-uninfected patients, the proportions of fungi (*P* < 0.001), viruses (*P* < 0.001), and Mycobacterium (*P* = 0.011) were significantly higher in HIV-infected patients, while the proportions of bacteria and atypical microorganisms (including *Mycoplasma*, Chlamydia, and *Legionella*) were not significantly different between the two groups.

A further comparison of lung microorganisms between HIV-infected and HIV-uninfected patients with pulmonary infection was performed by mNGS of BALF and is shown in Fig. 2B. The proportions of Pseudomonas aeruginosa (*P* = 0.023), Klebsiella pneumoniae (*P* < 0.001), Stenotrophomonas maltophilia (*P* < 0.001), and Chlamydia (*P* = 0.012) among HIV-infected patients were significantly lower than those observed among HIV-uninfected patients. The proportions of Tropheryma whipplei (*P* = 0.006), MTB (*P* = 0.018), Pneumocystis jirovecii (*P* < 0.001), *Talaromyces marneffei* (*P* < 0.001), cytomegalovirus (*P* < 0.001), and *Mycoplasma* (*P* = 0.049) were significantly higher in HIV-infected patients than in HIV-uninfected patients.

### Constituent ratio of bacteria detected by mNGS.

**FIG 3:**
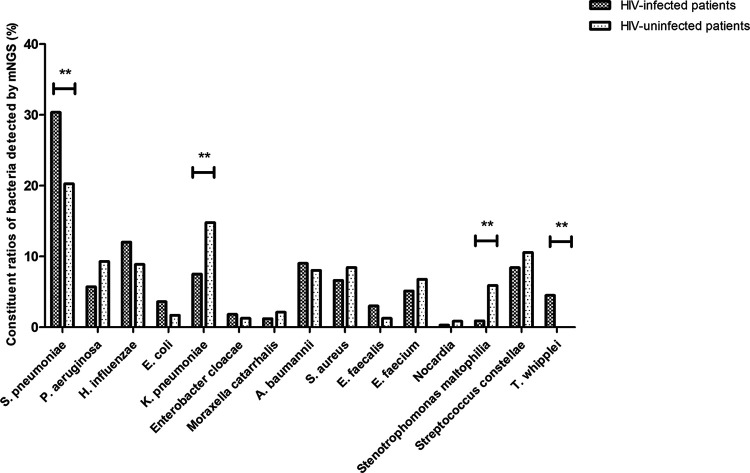
Comparison of constituent ratios of bacteria detected by mNGS of BALF between HIV-infected and HIV-uninfected patients; *, *P* < 0.05; **, *P* < 0.01.

As shown in Fig. 3, Streptococcus pneumoniae (30.33%), Haemophilus influenzae (12.01%), and Acinetobacter baumannii (9.01%) were more common in the bacteria spectrum among HIV-infected patients with pulmonary infection. Compared with HIV-uninfected patients, the constituent ratios of S. pneumoniae (*P* = 0.007) and T. whipplei (*P* = 0.002) were significantly higher in HIV-infected patients, while the constituent ratios of K. pneumoniae (*P* = 0.005) and Stenotrophomonas maltophilia (*P* = 0.001) were significantly lower in HIV-infected patients.

### Constituent ratios of Mycobacterium and fungi detected by mNGS.

**FIG 4:**
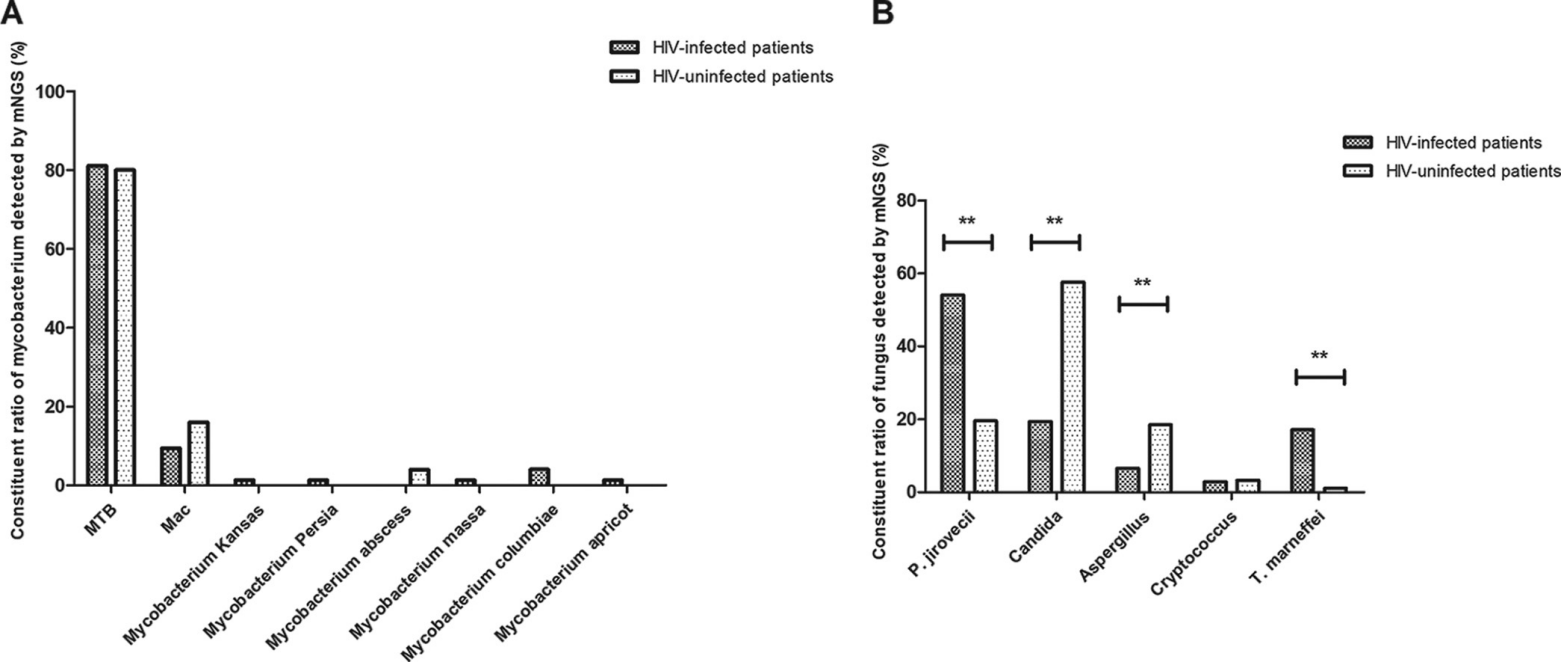
(A) Comparison of constituent ratios of Mycobacterium detected by mNGS of BALF between HIV-infected and HIV-uninfected patients. (B) Comparison of constituent ratios of fungi detected by mNGS of BALF between HIV-infected and HIV-uninfected patients; *, *P* < 0.05; **, *P* < 0.01.

As shown in Fig. 4A, MTB and Mycobacterium avium complex (Mac) were more common in the Mycobacterium spectrum detected by BALF mNGS in both HIV-infected and uninfected patients. However, the constituent ratios of different mycobacteria in the Mycobacterium spectrum were not significantly different between HIV-infected and uninfected patients.

As shown in Fig. 4B, *P. jirovecii* (54.08%), *Candida* (19.39%), and *T. marneffei* (17.14%) were more common in the fungal spectrum detected by BALF mNGS in HIV-infected patients. Compared with HIV-uninfected patients, the constituent ratios of *P. jirovecii* (*P* < 0.001) and *T. marneffei* (*P* < 0.001) were significantly higher, while the constituent ratios of C*andida* (*P* < 0.001) and Aspergillus (*P* < 0.001) were significantly lower in the fungal spectrum in HIV-infected patients.

### Effect of ART on lung microbiomes among HIV-infected patients.

**FIG 5:**
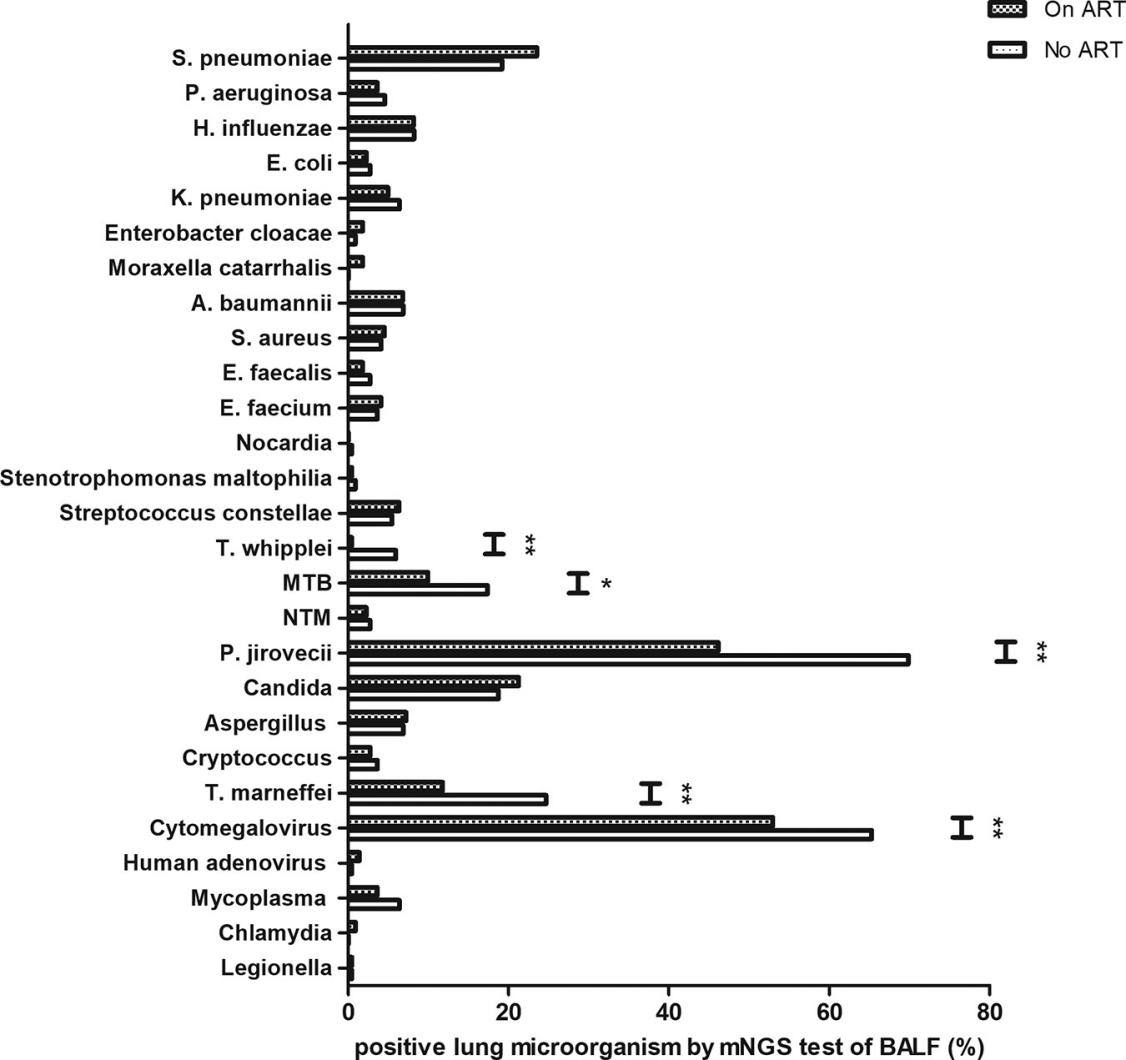
Comparison of lung microbiomes detected by mNGS of BALF between HIV-infected patients on ART and HIV-infected patients without ART; *, *P* < 0.05; **, *P* < 0.01.

Using mNGS of BALF, we further compared the lung microbiomes of individuals with pulmonary infections that were infected with HIV and on ART to those that were not treated with ART. The characteristics of the two groups are shown in Table S1 in the supplemental material. As shown in Fig. 5, among HIV-infected patients on ART, cytomegalovirus (52.9%), *P. jirovecii* (46.1%), and S. pneumoniae (23.5%) were the most commonly detected microorganisms, while *P. jirovecii* (69.8%), cytomegalovirus (65.3%), and *T. marneffei* (24.6%) were the most common microorganisms detected among HIV-infected patients without ART. In comparison to HIV-infected patients without ART, the proportions of T. whipplei (*P* = 0.001), MTB (*P* = 0.024), *P. jirovecii* (*P* < 0.001), *T. marneffei* (*P* < 0.001), and cytomegalovirus (*P* = 0.008) were significantly lower in HIV-infected patients on ART. The detection rates of common bacteria were not significantly different between the two groups.

## DISCUSSION

Understanding the lung microbiomes of HIV-infected patients with pulmonary infection will be helpful for making earlier and more accurate diagnoses and treatment regimens. Our study is the first to provide comprehensive information on the lung microbiomes of HIV-infected patients with pulmonary infection by using more sensitive mNGS of BALF and comparing with HIV-uninfected patients. We found significant differences in the lung microbiomes between HIV-infected and HIV-uninfected patients with pulmonary infection, and ART influenced the lung microbiome profile of HIV-infected patients with pulmonary infection, which provides more references for the clinic.

Currently, few studies have systematically described the spectrum of pulmonary infection among HIV-infected patients. A previous review summarized the etiology of pulmonary infections in HIV-infected patients and showed that bacterial pneumonia is the most frequent cause of pulmonary infections in HIV-infected patients, followed by pneumocystis pneumonia (PCP) and pulmonary tuberculosis (TB), with different incidences based on different geographical areas (1). However, our study showed that fungi (mainly PCP) and viruses (mainly cytomegalovirus), followed by bacteria, were the most common lung microbiomes among HIV-infected patients with pulmonary infection. Recently, in a cohort study of 82 HIV-infected patients admitted with a respiratory complaint, respiratory viruses were the most commonly identified respiratory pathogens, as determined by real-time reverse transcription PCR and NGS of nasopharyngeal swabs or BALF, more than bacterial and fungal pathogens combined (14). Improvements in diagnostic techniques such as mNGS have greatly increased the detection rate of viruses among HIV-infected patients with pulmonary infection.

In our study, the constituent ratios of S. pneumoniae in the bacteria spectrum were the highest in both HIV-infected and uninfected patients with pulmonary infection. Although the detection rate of S. pneumoniae between HIV-infected and uninfected patients was not significantly different, the constituent ratio of S. pneumoniae was significantly increased in the former group, suggesting a relatively higher risk of S. pneumoniae infection in HIV-infected patients with bacterial pneumonia. S. pneumoniae was reported to be the leading bacteria causing community-acquired pneumonia in HIV-infected patients (15–17), and people with HIV infection were at a greater risk of pneumococcal pneumonia than HIV-uninfected individuals (18, 19). An *in vivo* study found that HIV-infected individuals had a higher propensity for harboring S. pneumoniae within alveolar macrophages than HIV-uninfected individuals, and bacterial intracellular survival in alveolar macrophages was related to extracellular propagation of pneumococcal infection (20). Moreover, we found that the detection rate and constituent ratio of K. pneumoniae were significantly lower in HIV-infected patients than in HIV-uninfected patients, which may suggest that the risk of K. pneumoniae infection is lower in HIV-infected patients.

Our data found a significantly higher detection rate and constituent ratio of T. whipplei in BALF among HIV-infected patients than among HIV-uninfected patients. Compared with HIV-uninfected patients, a significantly higher burden of T. whipplei colonization in the lungs has been observed in HIV-infected patients (21). A study performed in HIV-infected patients from the United States reported that the prevalence of T. whipplei colonization in BALF and induced sputum samples was as high as 43.4% (22). However, pulmonary involvement of T. whipplei infection is rarely reported in HIV-infected patients (23, 24). The pathogenic mechanism of T. whipplei needs further exploration in the future.

In our study, the higher positive detection rate of MTB contributed to the significantly increased proportion of Mycobacterium among HIV-infected patients than among HIV-uninfected patients. Undoubtedly, the prevalence of MTB in HIV-infected individuals is obviously higher than that in HIV-uninfected individuals (1, 25). We also found that Mac was the most frequent nontuberculous mycobacteria (NTM) in the Mycobacterium spectrum among both HIV-infected and HIV-uninfected patients, which was consistent with the results of previous studies that showed that Mac was the most common NTM species in nontuberculous mycobacterial pulmonary disease in China (26, 27).

We observed that higher positive rates of *P. jirovecii* and *T. marneffei* contributed to the significantly higher proportion of fungi observed among HIV-infected patients than among HIV-uninfected patients, and the constituent ratios of *P. jirovecii* and *T. marneffei* in the fungal spectrum were significantly increased among HIV-infected patients versus among HIV-uninfected patients in China. Currently few studies have reported the positive rate of *T. marneffei* in BALF. A retrospective study conducted in HIV-infected patients with *T. marneffei* infection showed that the positive rate of *T. marneffei* in BALF was 97.6% (28). Compared with the other sites *in vivo*, the lung was the primary entry portal and had the highest burden of *T. marneffei* (29, 30). These data suggest that the high positive rate of pulmonary *T. marneffei* among HIV-infected patients was bound up with *T. marneffei* infection.

We found that the constituent ratios of C*andida* and Aspergillus were significantly decreased in the fungal spectrum among HIV-infected patients compared with HIV-uninfected patients. Previous studies conducted in HIV-uninfected patients with suspected pulmonary fugal infection and suspected pulmonary infection showed that *Candida* spp. were the most frequently detected fungal pathogen, following by *P. jirovecii* and Aspergillus (31, 32), which were consistent with our results. A study including 35,252 HIV-infected patients from a national HIV surveillance database estimated that the incidence of invasive aspergillosis was 3.5 cases per 1,000 person years (33). Another database including 38 million hospital diagnoses showed that aspergillosis was diagnosed in 0.43% of HIV-infected patients (34), together suggesting that aspergillosis was uncommon in HIV-infected patients. The depletion of neutrophils and macrophages may be more closely related to Aspergillus infection instead of the depletion of CD4^+^ T cells (35).

Our study showed that the proportions of T. whipplei, MTB, *P. jirovecii*, *T. marneffei*, and cytomegalovirus detected by BALF mNGS were significantly lower among HIV-infected patients on ART than among HIV-infected patients without ART, suggesting that ART could reduce the risk of pulmonary infection caused by T. whipplei, MTB, *P. jirovecii*, *T. marneffei*, and cytomegalovirus. Studies have reported that ART could reduce the burden of T. whipplei colonization in the lungs among HIV-infected patients (21, 36). However, we did not observe significant changes in the proportions of common bacteria among HIV-infected patients with pulmonary infection who were on ART. We speculated that ART may have a greater effect on pulmonary infection caused by nonbacterial pathogens, especially opportunistic pathogens, than on diseases caused by bacteria. Currently, few studies have reported a decreased incidence of pulmonary S. pneumoniae infection after ART among HIV-positive patients (37, 38), but several studies also showed no change in the incidence of pulmonary S. pneumoniae infection after ART (39, 40). Dysfunction of humoral immunity, IgA levels at mucosal surfaces, killing activity of mononuclear cells, and complement bactericidal activity may contribute to the sustained incidence of S. pneumoniae infection (39, 41, 42).

The strength of this study is that we provide a valuable reference for the etiology of pulmonary infection among HIV-infected patients by using a more sensitive mNGS test of BALF and providing more comprehensive information on the lung microorganisms of HIV-infected patients with pulmonary infection by comparing to those of HIV-uninfected patients. Our study also has some limitations. First, this study was performed in a single referral center and may not represent the etiology of pulmonary infection in general. However, we have a relatively large sample size compared to other studies. Second, despite the high sensitivity of mNGS, the absence of standardized interpretation for mNGS may potentially lead to false-positive or false-negative results. However, all the criteria for positive mNGS results in our study adhered to standards established by previous studies with comparable sequencing platforms and samples (8, 43). In terms of respiratory specimen types, our study focused on mNGS results from BALF rather than from upper respiratory tract specimens. Studies have reported that microorganism colonization in oral and upper respiratory tract specimens is usually greater than that in lower respiratory tract specimens, and the pathogenic microbial load in BALF specimens is typically higher and less affected by interference from commensals and human nucleic acids (44). In the future, more comprehensive and accurate standards need to be explored to interpret the pathogenicity of microorganisms detected by mNGS.

### Conclusion.

Significant differences in lung microbiomes, including bacteria, Mycobacterium, fungi, and viruses, exist between HIV-infected and uninfected patients with pulmonary infection, and ART influences the lung microbiomes among HIV-infected patients with pulmonary infection.

## MATERIALS AND METHODS

### Study participants.

Patients with pulmonary infection who had mNGS performed on BALF samples were retrospectively reviewed from the First Hospital of Changsha between January 2019 and June 2022. The exclusion criteria were as follows: (i) unknown HIV infection status, (ii) age of <18 years old, and (iii) pregnancy. Patients who met the following criteria were considered for pulmonary infection: (i) chest imaging suggestive of pulmonary infection; (ii) fever, cough, expectoration, dyspnea, or other respiratory symptoms, leukocytosis, or leukocytopenia; and (iii) determined by the consensus of two experienced senior clinicians based on clinical manifestation, laboratory tests, chest imaging, smear and culture of bacteria and fungi, 1,3-β-D glucan (G) assay (G test) and galactomannan (GM) assay, cytomegalovirus DNA PCR test, tuberculin skin test (TST), interferon-γ release assay (IGRA), acid-fast stain, GeneXpert, positive mNGS result of BALF, and response to antibiotic therapy.

### Data collection.

Clinical data were retrospectively obtained from medical records, and the following data were extracted: age, gender, HIV infection status, date of antiretroviral therapy (ART) initiation, date of BALF sample collection, antibiotic use within 3 months, immunosuppressive therapy (glucocorticoids and immunosuppressants) within 3 months, HIV load, lymphocyte count, CD4^+^ T lymphocyte count (CD4 count), and results of mNGS.

### BALF sample processing and DNA extraction.

For each participant enrolled, 1.5 to 3.5 mL of BALF was collected according to standard procedures. A 1.5-mL microcentrifuge tube with 0.6 mL of sample and 250 μL of 0.5-mm glass beads was attached to a horizontal platform on a vortex mixer and agitated vigorously at 2,800 to 3,200 rpm for 30 min. Lysozyme (7.2 μL; RT410-TA, Tiangen Biotech, Beijing, China) was added for the wall-breaking reaction. Then, 0.3 mL of sample was separated into a new 1.5-mL microcentrifuge tube, and DNA was extracted using the TIANamp Micro DNA kit (DP316, Tiangen Biotech), according to the manufacturer’s instructions.

### Sequencing and bioinformatic analysis.

DNA libraries were constructed through DNA fragmentation, end repair, adapter ligation, and PCR amplification. An Agilent 2100 was used for quality control of the DNA libraries. Quality libraries were pooled, and DNA Nanoball (DNB) was made and sequenced by the BGISEQ-50/MGISEQ-2000 platform. High-quality sequencing data were generated by removing low-quality reads, followed by computational subtraction of human host sequences mapped to the human reference genome (hg19) using Burrows-Wheeler alignment. The remaining data by removal of low-complexity reads were classified by simultaneously aligning to Pathogens Metagenomics Database (PMDB), consisting of bacteria, fungi, viruses, and parasites. The classification reference databases were downloaded from NCBI (ftp://ftp.ncbi.nlm.nih.gov/genomes/). RefSeq contains 4,945 whole-genome sequences of viral taxa, 6,350 bacterial genomes or scaffolds, 1,064 fungi related to human infection, and 234 parasites associated with human diseases.

### Criteria for positive mNGS results.

The criteria for positive mNGS results were defined as follows (8, 43). (i) There is evidence of pulmonary pathogenicity supported by the literature. (ii) For bacteria (except for mycobacteria), viruses, and parasites, the coverage rate of a microbe (species level) scored 10-fold greater than that of other microbes of the same type. (iii) For Mycobacterium tuberculosis (MTB), at least 1 read mapped to the species or genus level, and for nontuberculous mycobacteria (NTM), the reads mapped to the species or genus level rank in the top 10 in the bacteria list. (iv) For fungi, the coverage rate of a microbe (species level) scored 5-fold greater than that of other fungi. Oral commensals, including Streptococcus infantis, Oribacterium parvum, Prevotella pallens, Kingella oralis, Mogibacterium timidum, Treponema maltophilum, Dialister invisus, Streptococcus parasanguinis, Rothia dentocariosa, Campylobacter showae, Actinomyces johnsonii, Johnsonella ignava, Cardiobacterium hominis, Scardovia wiggsiae, Desulfomicrobium orale, Fretibacterium fastidiosum, Actinomyces gerencseriae, coagulase-negative staphylococci, *Neisseria*, etc. are normally parasitic in the human oropharynx and are not considered to cause pulmonary infection according to the literature and previous studies (4, 32, 45). Therefore, they were not considered positive regardless of their reads.

It should be noted that the identification of whether a microorganism was infected, colonized, or contaminated was based on the comprehensive assessment of patients’ clinical manifestations, immune status, underlying diseases, chest imaging, mNGS results, other auxiliary examinations, response to antibiotic therapy, and clinical experts’ judgments.

### Ethics approvals.

This study was approved by the Institutional Ethics Committee of the First Hospital of Changsha (202128). All subjects voluntarily participated in the study and signed the informed consent form.

### Statistical analysis.

Statistical analyses and graphing were performed using SPSS 21.0 and GraphPad Prism 5.0. Kolmogorov-Smirnov tests were used to examine whether the measurement data conformed to the normal distribution. Continuous variables were denoted as medians with the 25th to 75th interquartile range (IQR), and categorical variables were denoted as proportion (%). Continuous variables that conformed to the normal distribution were analyzed by group *t* test and vice versa were analyzed by nonparametric rank-sum test. A chi-square test was used to compare the count data between the groups. The comparisons of lung microorganisms between two groups were performed by Pearson chi-square test or Fisher’s exact test. A *P* value of <0.05 was considered significant.

### Data availability.

The data sets used and analyzed during the current study are available from the corresponding authors upon reasonable request. Sequencing data that support the findings of this study have been deposited in NCBI SRA and can be accessed with the BioProject ID PRJNA977832.
