# Supplementary material for: Microbiomes Detected by Bronchoalveolar Lavage Fluid Metagenomic Next-Generation Sequencing among HIV-Infected and Uninfected Patients with Pulmonary Infection
Source: Microbiol Spectr. 2023 Jul 12;11(4):e00005-23. doi: 10.1128/spectrum.00005-23 (PMC10434007; doi:10.1128/spectrum.00005-23)
Supplement: Supplemental file 1 — Table S1. Download spectrum.00005-23-s0001.docx, DOCX file, 0.01 MB [file spectrum.00005-23-s0001.docx]

Supplementary Table 1. Characteristics of HIV-infected patients with pulmonary infection, comparing those on ART to those not receiving ART.

|  | HIV-infected patients on ART (n=221) | HIV-uninfected patients without ART (n=219) | p valune |
| --- | --- | --- | --- |
| Age [years, median (IQR)]  Male, n(%）  Duration of ART [days, median (IQR)]  Antibiotic using within 3 months, n(%)  Immunosuppressive therapy use within  3 months, n(%)  HIV viral load [copies/ml, median (IQR)]  Lymphocyte counts [/ul, median(IQR)]  CD4 count [/ul, median (IQR)] | 50 (37~57)  189 (85.5)  44 (14-738)  207 (93.6)  78 (35.3)  33,800 (226~219,000)  810 (540~1325)  88 (34~220) | 48 (36~57)  180 (82.2)  /  212 (96.8)  89 (40.6)  324,000 (127,000-816,500)  630 (400~947)  36 (15-84) | 0.572  0.343  0.187  0.248  <0.001  0.003  <0.001 |
